# Malignant pleural mesothelioma and mesothelial hyperplasia: A new molecular tool for the differential diagnosis

**DOI:** 10.18632/oncotarget.13174

**Published:** 2016-07-11

**Authors:** Rossella Bruno, Greta Alì, Riccardo Giannini, Agnese Proietti, Marco Lucchi, Antonio Chella, Franca Melfi, Alfredo Mussi, Gabriella Fontanini

**Affiliations:** ^1^ Department of Surgical, Medical, Molecular Pathology and Critical Area, University of Pisa, Pisa, Italy; ^2^ Division of Pathological Anatomy, University Hospital of Pisa, Pisa, Italy; ^3^ Division of Thoracic Surgery, University Hospital of Pisa, Pisa, Italy; ^4^ Division of Pneumology, University Hospital of Pisa, Pisa, Italy; ^5^ Program of Pleuropulmonary Pathology, University Hospital of Pisa, Pisa, Italy

**Keywords:** malignant pleural mesothelioma, mesothelial hyperplasia, differential diagnosis, gene profiles, classification models

## Abstract

Malignant pleural mesothelioma (MPM) is a rare asbestos related cancer, aggressive and unresponsive to therapies. Histological examination of pleural lesions is the gold standard of MPM diagnosis, although it is sometimes hard to discriminate the epithelioid type of MPM from benign mesothelial hyperplasia (MH).

This work aims to define a new molecular tool for the differential diagnosis of MPM, using the expression profile of 117 genes deregulated in this tumour.

The gene expression analysis was performed by nanoString System on tumour tissues from 36 epithelioid MPM and 17 MH patients, and on 14 mesothelial pleural samples analysed in a blind way. Data analysis included raw nanoString data normalization, unsupervised cluster analysis by Pearson correlation, non-parametric Mann Whitney U-test and molecular classification by the Uncorrelated Shrunken Centroid (USC) Algorithm.

The Mann-Whitney U-test found 35 genes upregulated and 31 downregulated in MPM. The unsupervised cluster analysis revealed two clusters, one composed only of MPM and one only of MH samples, thus revealing class-specific gene profiles. The Uncorrelated Shrunken Centroid algorithm identified two classifiers, one including 22 genes and the other 40 genes, able to properly classify all the samples as benign or malignant using gene expression data; both classifiers were also able to correctly determine, in a blind analysis, the diagnostic categories of all the 14 unknown samples.

In conclusion we delineated a diagnostic tool combining molecular data (gene expression) and computational analysis (USC algorithm), which can be applied in the clinical practice for the differential diagnosis of MPM.

## INTRODUCTION

Malignant pleural mesothelioma (MPM) is a highly aggressive and relatively rare tumour originating from mesothelial cells showing a diffuse pattern of growth over the pleural surface. MPM is an asbestos related cancer and its incidence in Europe is about 20 per million, with a great intercountry variation and an incidence peak expected around 2020 according to the widespread exposure to asbestos in environmental and occupational situations [1, 2]. The long-term survival rate of MPM patients is poor and, presently, there is no satisfactory treatment for MPM [3–6].

MPM is an heterogeneous tumour, including three main histological subtypes: epithelioid (60-80%), sarcomatoid (< 10%) and mixed (10-15%) [7]. Cancerogenic mechanisms of MPM are still largely unknown and there are only few biomarkers available for diagnosis, prognosis and treatment.

The histological diagnosis of MPM is mainly based on the histological analysis of pleural lesions [8], but the morphological criteria of a lesion are not always clear and sometimes the analysis is challenging. One of the principal diagnostic issues is the separation of the epithelioid type of MPM from reactive mesothelial hyperplasia (MH) [9]. MH may be extremely florid mimicking mesothelioma in the context of a wide variety of diseases, such as infections, collagen vascular disease, pulmonary infarction and pneumothorax. The major criteria for distinguishing malignant mesothelioma from reactive mesothelial proliferations are based on the evaluation of cellularity, papillae, growth pattern, zonation, vascularity and stromal invasion, with the latter considered the most reliable indicator of malignancy [7, 10, 11]. In several cases the application of the previously reported criteria may be difficult according to the size of the biopsy specimen, the sampling, the tangential cuts and the entrapment of mesothelial cells [11].

Nowadays the status of MPM diagnostic biomarkers is not completely satisfactory. The protein mesothelin has been described as an absolutely promising biomarker, because its altered levels in serum and pleural fluid are usually associated with MPM, however, in spite of its high specificity, its sensitivity is low [12, 13]. In addition, the deletion of *CDKN2A* and *BAP1* are the most common genetic alterations in MPM [14] and for this reason they have been suggested as diagnostic and prognostic markers. The FISH analysis of *CDKN2A* and the immunohistochemistry analysis of BAP1 could enable the differential diagnosis of benign and malignant mesothelial proliferations, either alone or together [15]. The analyses of BAP1 and *CDKN2A* were shown to be highly specific for malignant pleural mesothelioma, both on tissue samples and pleural effusions, but their low sensitivity limits their clinical utility, as a negative result is not able to rule out a diagnosis of malignant mesothelioma [16, 17].

Moreover, in the last few years some methods combining supervised data mining and molecular analysis have been applied to classification tasks in mesothelioma. In 2014 Parodi et al. built a molecular classifier based on the concentration of three tumour markers (CEA, CYFRA 21-1 and SMRP) in pleural fluid, by using the Logic Learning Machine (LLM). They used the LLM model to classify malignant mesothelioma, pleural metastases from other tumours and benign pleural diseases, reporting a classification accuracy of 77,5% [18]. In 2015 Tosun et al. developed a diagnostic model based on the nuclear chromatin distribution from digital images of mesothelial cells in effusion cytology specimens and on the k-nearest neighbourhood algorithm. They analysed 34 cases obtaining a 100% accurate prediction in the discrimination of malignant from benign mesothelial proliferations [19]. Despite the promising results of the above mentioned studies, a definitive diagnostic tool has yet to be identified.

Recently, different studies have revealed gene pathways specifically deregulated in MPM tissues with a crucial role in cancer development and progression [20–25]. The majority of the deregulated genes in MPM belong to the following pathways: angiogenesis, cell adhesion, p53 signalling, integrin signalling, MAPK signalling, apoptosis and cell cycle regulation [26–32]. Although there is a clear implication of these genes in cancer, sensitive markers for MPM are still missing.

According to the high heterogeneity of MPM it is likely that it might not be sufficient to have only one or only a few genes for the differential diagnosis. For this reason, we investigated how the deregulated genes work together in discriminating malignant from benign pleural proliferations. Taking on board the latest papers about MPM genes we designed an nCounter custom codeset, consisting of 117 genes (Table 1), in order to perform a gene expression profiling of epithelioid MPM and MH samples, using nanoString technologies. Furthermore, we used the uncorrelated shrunken centroid (USC) classification algorithm to delineate molecular classifiers, which could be directly applied in the differential diagnosis of MPM and MH.

**Table 1:** nCounter custom codeset

nCounter custom codeset	
FUNCTIONAL ANNOTATION	GENES
ACETYLATION	*ACSL1, ASS1, DNMT1, EEF2, EIF4G1, GNAQ, SMARCA4, TNPO2, TOP2A, XPOT*
CELL ADHESION	*BMP1, CD44, CDH1, CDH11, CLDN15, COL16A1, CTNNA1, CXADR, EGFR, FN1, ITGA3, ITGA4, ITGA5, ITGA7, ITGAM, ITGB4, LAMA3, LAMC1, LGALS3BP, MSLN, NME2, PECAM1, SELE, THBS2, VWF*
CELL CYCLE	*AURKA, BIRC5, BUB1, CCNB1, CCNB2, CCNO, CDK1, CDK4, CDK7, CDKN2A, CDKN2B, CENPF, CHEK1, EGFR, FANCI, MAD2L1, MCM2, MCM4, MK167, NDC80, PCNA, PLK1, PLK2, RAD21, TACC1, TUBB2B*
DNA MODIFICATOR	*DNMT1, DNMT3*
ECM RECEPTOR INTERACTION	*CD44, COL1A1, COL4A2, FN1, ITGA3, ITGA4, ITGA5, ITGA7, ITGB4, LAMA3, LAMC1, SOD1, THBS2, VWF*
EXTRACELLULAR MATRIX	*ADAMTS8, CD44, COL16A1, COL1A1, COL4A2, FN1, HEG1, LAMA3, LAMC1, LGALS3, LGALS3BP, MMP1, MMP10, MMP12, MMP14, MMP3, MMP7, MMP9, SFRP1, SOD1, TIMP3, VEGFA, VWF*
FOCAL ADHESION	*CAV1, COL1A1, COL4A2, EGFR, FN1, ITGA3, ITGA4, ITGA5, ITGA7, ITGB4, LAMA3, LAMC1, PAK4, PDGFRB, PIK3CA, THBS2, VEGFA, VWF*
INTRACELLULAR NON MEMBRANE BOUNDED ORGANELLE	*DNMT3A, DSP, JUNB, MICAL2, MYH11, SMARCA4, TERT, TOP2A, TPPP*
METAL ION BINDING	*GALNT7, PKM2, PTGIS, SDHB*
NUCLEOTIDE BINDING	*ACSL1, ADCY4, ASS1, EEF2, EGR3, EMX2, GNAQ, JUNB, MYH11, PPARA, SMARCA4, TERT, TOP2A, UBE2T*
NUCLEUS	*DNMT3A, DNTM1, EGR3, EMX2, JUNB, PPARA, SMARCA4, TERT, TNPO2, TOP2A, TPPP, XPOT*
PATHWAYS IN CANCER	*BIRC5, CDH1, CDKN2A, CDKN2B, COL4A2, CTNNA1, EGFR, FGF2, FN1, GLI1, GLI2, ITGA3, LAMA3, LAMP1, MMP1, MMP9, PDGFRB, PIK3CA, PTGS2, TGFBR2, VEGFA*
REGULATION OF CELL PROLIFERATION	*ADAMTS8, BAP1, CAV1, CD274, CDK4, CDKN2A, CDKN2B, CHEK1, CXADR, EGFR, ESR2, FGF2, GLI1, GLI2, IFITM1, JAG1, KRT5, LAMC1, MAGED1, MMP12, MMP7, NF2, NME2, NOTCH1, PDGFRB, PTGS2, SERPINE1, TGFR2, VEGFA*
SIGNALING	*ADAMTS8, BMP1, CD274, CD44, CDH1, CDH11, CFB, COL16A1, COL1A1, COL4A2, CXADR, EGFR, FN1, HEG1, ITGA3, ITGA4, ITGA5, ITGA7, ITGAM, ITGB4, JAG1, LAMA3, LAMC1, LGALS3BP, MMP1, MMP10, MMP14, MMP3, MMP7, MMP9, MSLN, NMU, NOTCH1, PAPPA, PDCD1, PDGFRB, PECAM1, SDC1, SELE, SERPINE1, SULF1, THBS2, TIMP3, VEGFA, VWF*
REFERENCE GENES	*CLTC, GAPDH, GUSB, HPRT1, PGK1, TUBB*

Genes included in the nanoString custom panel. Functional annotation of the selected genes was obtained from the Database for Annotation, Visualization and Integrated Discovery (DAVID-david.ncifcrf.gov).

## RESULTS

### nanoString data normalization

Gene expression profiling using nanoString technology was performed on 36 epithelioid MPM samples and 17 MH samples. The raw data normalization was executed, as described in materials and methods section, in 2 steps, and 11 MPM samples and 2 MH samples were excluded from further statistical analysis on the basis of the biological normalization factor, thus indicating an mRNA input of poor quality. The samples which failed the biological normalization were obtained from archive materials older than two years.

### Hierarchical unsupervised clustering analysis and Mann Whitney U-test

**Figure 1:**
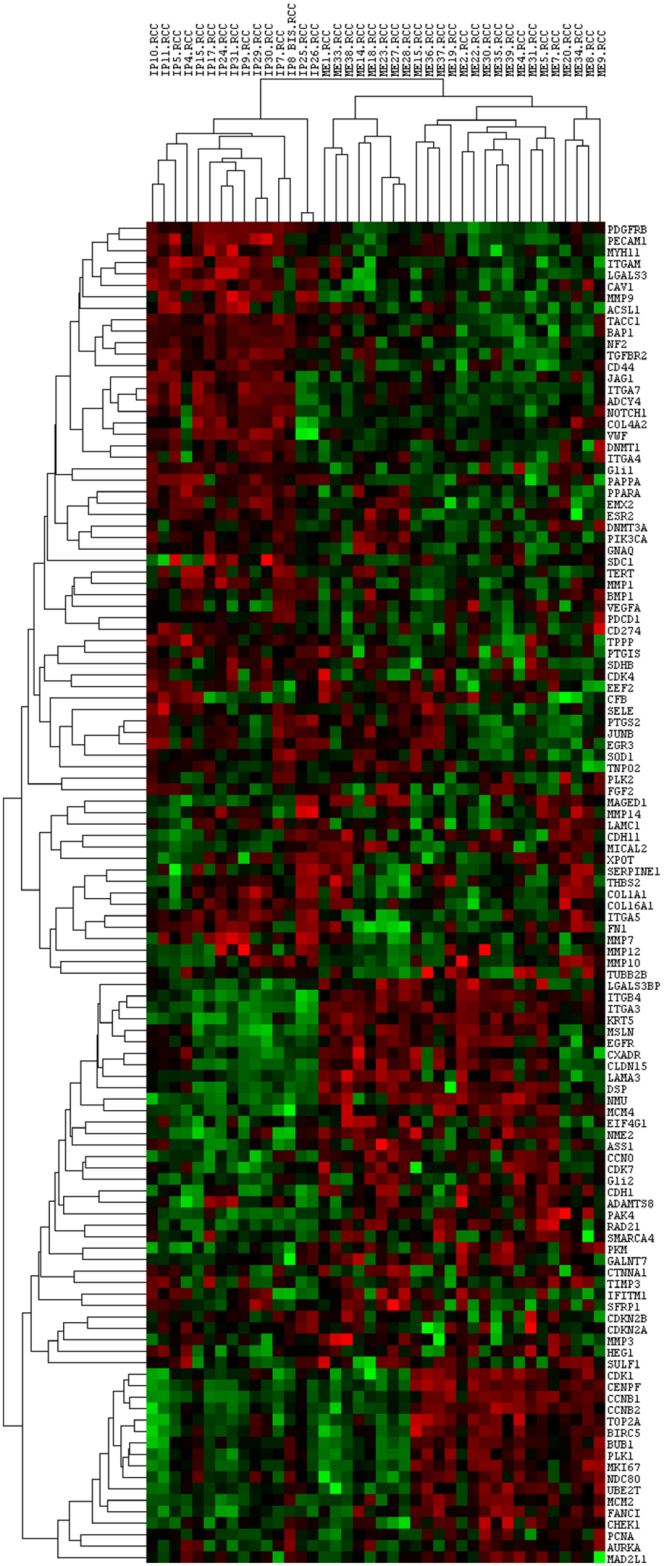
Unsupervised cluster analysis of all the analysed genes and samples Each column represents a single sample and each row a single gene. *IP*: reactive mesothelial hyperplasia (MH); *ME*: Epithelioid mesothelioma (MPM); *.RCC*: file extension. Red indicates a high level of expression relative to the mean expression, and green indicates a low level of expression relative to the mean expression.

In order to model gene expression profiles of benign and malignant pleural lesions, an unsupervised hierarchical clustering analysis (HCA) of Pearson correlation similarity matrix was performed on genes and samples, 25 MPM and 15 MH. Figure 1 shows the unsupervised cluster from the 117 genes panel: the gene expression profile of epithelioid MPM differed from the one of MH, indeed all the malignant samples are grouped in a cluster (Correlation: 0,15) and all the benign in another one (Correlation: 0,28).

When comparing gene expression with status condition: a) 25 genes resulted upregulated and 18 resulted downregulated in MPM, using a p-value lower than 0.005; while, b) 35 genes resulted upregulated and 31 resulted downregulated in MPM using a p-value lower than 0.05. Fifty-one genes did not result statistically deregulated in MPM (Table 2).

**Table 2:** Differentially expressed genes between MH and MPM

UP REGULATED GENES IN MPM			DOWN REGULATED GENES IN MPM		
*GENES*	Z-VALUE	P-VALUE	*GENES*	Z-VALUE	P-VALUE
*ITGB4*	-4.70564	0.000003	*LGALS3*	4.79643	0.000002
*ITGA3*	-4.67538	0.000003	*PDGFRB*	4.61486	0.000004
*MCM4*	-4.52408	0.000006	*ITGAM*	4.37277	0.000012
*KRT5*	-4.46355	0.000008	*PECAM1*	4.25172	0.000021
*NMU*	-4.31461	0.000016	*CAV1*	4.16094	0.000032
*FANCI*	-4.22146	0.000024	*MMP9*	3.97959	0.000069
*CCNB1*	-4.07016	0.000047	*NF2*	3.82806	0.000129
*DSP*	-3.70702	0.000210	*PAPPA*	3.70702	0.000210
*RAD21*	-3.64650	0.000266	*ITGA5*	3.67676	0.000236
*CENPF*	-3.58597	0.000336	*TACC1*	3.67676	0.000236
*TOP2A*	-3.55571	0.000377	*MYH11*	3.49538	0.000473
*MCM2*	-3.52545	0.000423	*CD44*	3.40440	0.000663
*PAK4*	-3.52545	0.000423	*PPARA*	3.28336	0.001026
*EGFR*	-3.40440	0.000663	*ACSL1*	3.13205	0.001736
*MSLN*	-3.40440	0.000663	*TGFBR2*	3.10179	0.001924
*CCNB2*	-3.34388	0.000826	*BAP1*	3.07153	0.002130
*CLDN15*	-3.28336	0.001026	*VWF*	3.04143	0.002355
*CCNO*	-3.25551	0.001132	*MMP1*	3.02832	0.002459
*BIRC5*	-3.25328	0.001141	*FN1*	2.70839	0.006761
*CDK7*	-3.16231	0.001565	*ITGA7*	2.70839	0.006761
*CDH1*	-3.13222	0.001735	*ADCY4*	2.67813	0.007404
*NME2*	-3.07153	0.002130	*MMP7*	2.62642	0.008629
*LGALS3BP*	-2.98075	0.002876	*COL1A1*	2.58735	0.009672
*PKM*	-2.92022	0.003498	*NOTCH1*	2.52682	0.011510
*MKI67*	-2.85970	0.004241	*TPPP*	2.49930	0.012444
*BUB1*	-2.79918	0.005124	*JAG1*	2.37552	0.017525
*PLK1*	-2.58735	0.009672	*DNMT1*	2.13343	0.032890
*CDK1*	-2.55709	0.010556	*EGR3*	2.13343	0.032890
*CXADR*	-2.52682	0.011510	*EMX2*	2.04893	0.040470
*AURKA*	-2.49656	0.012541	*Gli1*	2.03705	0.041646
*UBE2T*	-2.43604	0.014850	*SOD1*	2.01238	0.044181
*Gli2*	-2.40578	0.016139	*PTGS2*	1.89134	0.058580
*LAMA3*	-2.31500	0.020614	*ESR2*	1.81249	0.069911
*CHEK1*	-2.13343	0.032890	*SELE*	1.78203	0.074745
*NDC80*	-2.13343	0.032890	*JUNB*	1.52820	0.126464
*MICAL2*	-1.95186	0.050956	*COL16A1*	1.37689	0.168546
*MMP3*	-1.93918	0.052480	*PTGIS*	1.31637	0.188051
*SMARCA4*	-1.92160	0.054657	*SDHB*	1.31637	0.188051
*ASS1*	-1.89134	0.058580	*EEF2*	1.28611	0.198406
*GALNT7*	-1.83081	0.067129	*COL4A2*	1.16506	0.243994
*MAGED1*	-1.83081	0.067129	*ITGA4*	1.16506	0.243994
*PCNA*	-1.83081	0.067129	*PIK3CA*	1.13480	0.256459
*EIF4G1*	-1.77029	0.076680	*MMP12*	1.04605	0.295541
*CDH11*	-1.67951	0.093054	*SDC1*	1.01376	0.310700
*MAD2L1*	-1.37689	0.168546	*BMP1*	0.98349	0.325365
*HEG1*	-1.01376	0.310700	*TUBB2B*	0.89276	0.371987
*ADAMTS8*	-0.91104	0.362276	*TERT*	0.82029	0.412050
*PLK2*	-0.83219	0.405304	*CD274*	0.77167	0.440313
*VEGFA*	-0.71114	0.476997	*CDK4*	0.77167	0.440313
*CTNNA1*	-0.62036	0.535022	*MMP14*	0.77167	0.440313
*CFB*	-0.59010	0.555126	*GNAQ*	0.74140	0.458449
*TIMP3*	-0.52957	0.596408	*THBS2*	0.71114	0.476997
*TNPO2*	-0.43879	0.660814	*PDCD1*	0.68092	0.495924
*IFITM1*	-0.40853	0.682886	*CDKN2B*	0.65062	0.515293
*MMP10*	-0.28336	0.776901	*SERPINE1*	0.46905	0.639033
*DNMT3A*	-0.25722	0.797008	*SFRP1*	0.37827	0.705232
*XPOT*	-0.25722	0.797008	*FGF2*	0.28748	0.773743
*SULF1*	-0.13618	0.891682	*LAMC1*	0.25722	0.797008
			*CDKN2A*	0.09096	0.927526

Up and downregulated genes in MPM, reported according to the results of Mann-Whitney U-test.

Considering that the average age of MPM and MH patients was different, a Spearman's correlation test was executed to evaluate the influence of age on gene expression either considering all the samples together and MH and MPM groups separately. None of the analysed genes showed a strong and statistically significant correlation with age.

**Figure 2:**
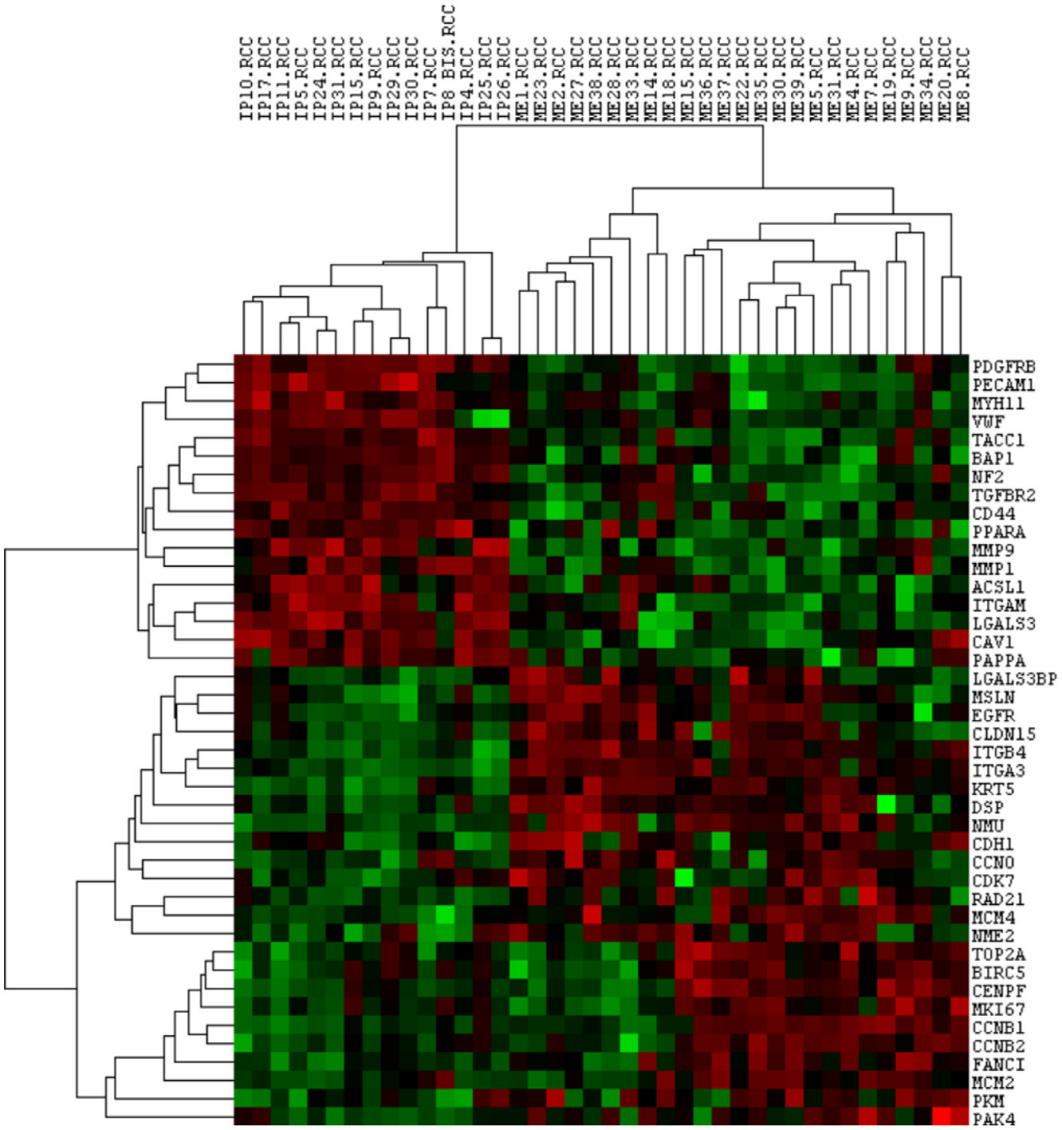
Unsupervised cluster analysis of statistically deregulated genes and all samples Each column represents a single sample and each row a single gene. *IP*: reactive mesothelial hyperplasia (MH); *ME*: Epithelioid mesothelioma (MPM); *.RCC*: file extension. Red indicates a high level of expression relative to the mean expression, and green indicates a low level of expression relative to the mean expression.

An unsupervised cluster analysis was also performed after we filtered for the differentially expressed genes (p-value < 0.005), obtaining a more evident and clear difference among the two groups (MH correlation: 0.56; MPM correlation: 0.29), as observed in Figure 2.

**Figure 3:**
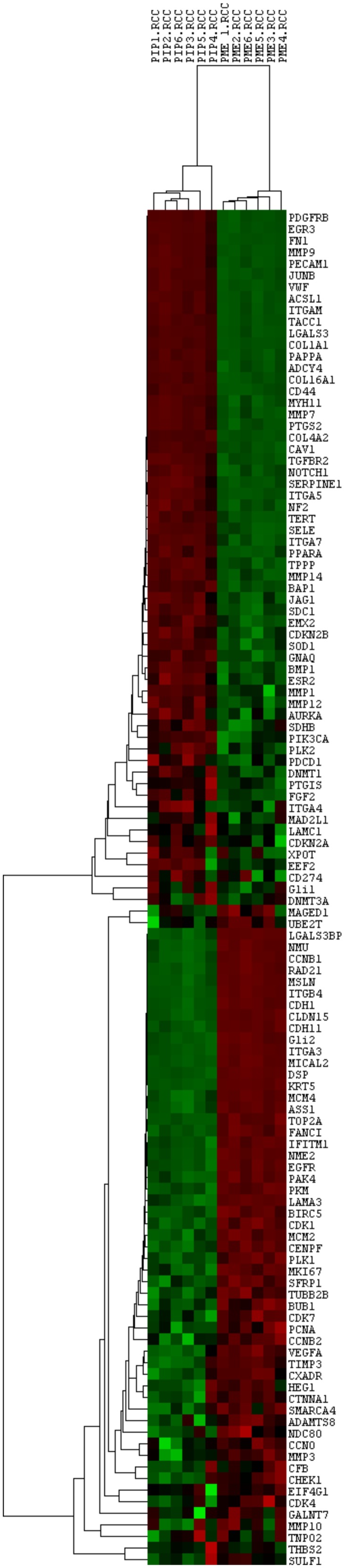
Unsupervised cluster analysis of all the analysed genes and the control pools Each column represents a single sample and each row a single gene. *PIP*: pool of reactive mesothelial hyperplasia samples; *PME*: pool of epithelioid mesothelioma samples; *.RCC*: file extension. Red indicates a high level of expression relative to the mean expression, and green indicates a low level of expression relative to the mean expression.

Moreover, to assess the reproducibility of nanoString System, a pool of MPM samples and a pool of MH samples were analysed in each experiment. Their counts were normalized and evaluated by an unsupervised cluster analysis using Pearson Correlation showing no difference among the same samples analysed in the different experiments (Figure 3).

### USC results

In the training phase a dataset composed of the normalized nanoString counts of all 117 genes was used and as training samples the entire cohort of 25 MPM and 15 MH was considered.

During the training step the system selected, on the basis of gene expression data, the best predictive genes specific for malignant and benign classes and gave two classification models as an output, namely Classifier 1 (22 genes) and Classifier 2 (40 genes). In the training set these classifiers were both able to predict the diagnostic category of a tissue with a classification error equal to 0 (Table 3). The USC parameters of the identified classifiers can be seen in Table 3.

**Table 3:** USC training results

MOLECULAR CLASSIFIERS		
	MODEL 1	MODEL 2
#Mistakes	0	0
Delta	1	0.5
Rho	0.7	0.7
Average Genes	18	34
Predictive Genes	*ASS1, BAP1, CAV1, CCNB1, CD44, CDH1, EGR3, FN1, ITGA3, KRT5, LAMA3, LGALS3, MICAL2, MMP9, MYH11, NME2, NMU, PAPPA, PECAM1, PKM, RAD21, TGFBR2*	ASS1, BAP1, CAV1, CCNB1, CD44, CDH1, CDH11, COL4A2, CTNNA1, CXADR, EEF2, EGR3, EIF4G1, FANCI, FN1, GALNT7, GLI2, HEG1, IFITM1, ITGA3, KRT5, LAMA3, LGALS3, MAGED1, MICAL2, MMP9, MYH11, NME2, NMU, PAK4, PAPPA, PECAM1, PKM, PTGS2, RAD21, SDC1, SMARCA4, TGFBR2, TOP2A, VEGFA

USC classifiers and parameters: *#Mistakes*: number of classification mistakes in the training phase; *Delta*: shrunken threshold; *Rho*: correlation threshold; *Average genes*: average number of genes, which are selected among the predictive ones, used for classification; *Predictive genes*: genes included in each classifier, selected among the 117 gave as input

In the test phase the molecular profiles of the 14 pleural mesothelial lesions analysed in a blind way were given to the USC algorithm as an input, which classified samples as epithelioid MPM or MH using Classifier 1 and separately Classifier 2.

Both the classifiers gave the same results: 5 samples resulted MH and 9 MPM. At the end of the test phase we compared USC results with histological diagnoses and all samples had been correctly classified as MH or MPM by our molecular models (Table 4).

**Table 4:** USC test results

	CLASSIFIER 1		CLASSIFIER 2		
SAMPLE	MOLECULAR CLASS	DISCRIMINANT SCORE	MOLECULAR CLASS	DISCRIMINANT SCORE	HISTOLOGICAL CLASS
1	MPM	28.07	MPM	41.57	MPM
2	MPM	93.44	MPM	115.4	MPM
3	MH	62.97	MH	160.5	MH
4	MPM	7.54	MPM	11.83	MPM
5	MH	8.6	MH	12.47	MH
6	MPM	12	MPM	16.75	MPM
7	MPM	29.05	MPM	37.11	MPM
8	MH	30.97	MH	34.39	MH
9	MH	52.15	MH	51.91	MH
10	MPM	8.37	MPM	15.91	MPM
11	MPM	11.42	MPM	14.06	MPM
12	MPM	10.12	MPM	16.3	MPM
13	MPM	10.46	MPM	15.82	MPM
14	MH	10.99	MH	13.84	MH

## DISCUSSION

The differential diagnosis of epithelioid MPM and MH has always been a discussed topic. Nowadays the diagnosis of MPM is mainly based on the histological analysis of pleural lesions and the most robust criterion for malignancy is the presence of stromal invasion [11]. The morphological examination of a lesion does not always lead to a conclusive diagnosis, particularly in cases of biopsies of mesothelial proliferations confined to the pleural surface and in case of cytological specimens [9].

In this study we identified a new molecular tool which combines molecular data and computational analysis to classify a mesothelial proliferation as benign (reactive hyperplasia) or as malignant (epithelioid MPM).

The approach that we have proposed includes the expression analysis of 117 genes deregulated in MPM, using the high sensitive and innovative nanoString system, and a computational elaboration of data by the USC classification algorithm.

We used nanoString System rather than the other available techniques because it allows direct counting of mRNA molecules, without any retro-transcription steps, so the potential errors associated with multiple qPCR assays are avoided. Moreover, this method requires a total amount of RNA as low as 150 ng, which can be easily obtained from formalin-fixed and paraffin-embedded (FFPE) samples. Indeed, a sufficient yield of RNA was obtained from all the samples analysed in this study. Furthermore, the 13 cases having a poor quality RNA were obtained from archive materials older than 2 years and none of the more recent samples failed the analysis, so this system is adequate for the analysis of FFPE specimens from pleural biopsies.

Firstly, the results of the unsupervised cluster analysis of all genes and samples revealed the ability of the whole panel to correctly group malignant and benign pleural tissues.

Then, in order to make our approach directly reliable for the clinical application we used the USC classification algorithm to objectively predict, on the basis of gene expression data, the diagnostic category of a sample. The decision to use the USC algorithm was due to the fact that it removes highly correlated genes and it does not require *a priori* assumptions, so normalized nanoString data from all of the genes could be directly used for computational classification, without any further manipulation.

The USC identified two classifiers, one which had 22 genes and the other 40 genes out of the initial 117, and both of these were able to classify samples as benign or as malignant without any errors. Some of the USC predictive genes, included in the classifiers, resulted also statistically deregulated in MPM in comparison to MH. The fact that not all the statistically deregulated genes have been included in the classifiers is probably due to the removal of highly correlated genes by the algorithm.

We could not calculate positive and negative predictive values of the identified classification models because of the small number of analysed samples. However, we achieved a 100% predictive accuracy from 40 FFPE samples, whose histological diagnosis was known before the test, and from 14 FFPE samples analysed in a blind test, whose histological classification was revealed only at the end of the computational analysis.

These results lead us to believe that the analysis of a larger series of samples may confirm the high specificity and sensitivity of these classifiers and might determine which one is better.

The USC algorithm applied to gene expression data could really improve the current diagnostic methods. Our analysis system has been shown to be highly reproducible, reliable and potentially appropriate for clinical purpose. Indeed, from a technical point of view we assessed the reproducibility of nanoString by repeating the analysis of a pool of benign and a pool of malignant samples in each experiment, and we reported an extremely low inter assays variability.

In addition, we validated the statistical deregulation of 66 genes out of the selected 117, among which there were several well-known mesothelioma genes, such as *MSLN, BAP1*, and *NF2* [13, 33, 34]. Most of the deregulated genes belong to signalling pathways that could drive the development of new targeted therapies, such as *Gli2*, which belong to the Hedgehog pathway, whose inhibition has been reported to suppress cell growth dramatically both in vitro and in vivo, so targeting this pathway could constitute a new effective treatment approach [30]. Furthermore, we confirmed the downregulation of *ITGA7*, which was reported to be epigenetically deregulated in MPM and suggested as therapeutic and prognostic marker [35], and the downregulation of *NF2*, altered in almost half of MPM tumours with an important prognostic impact [34].

Interestingly, according to Melaiu and collaborators in their meta-analysis [24], *CDKN2A* did not result statistically deregulated in MPM in comparison with MH. We also found a slight up regulation of *ASS1*, which was described to influence the sensitivity to chemotherapy. The up or down regulation of *ASS1* in MPM is quite controversial, many scientists considered MPM an *ASS1* lacking tumour, however, others reported an upregulation in this tumour [36, 37].

The reported gene expression data are certainly useful from a biological point of view, suggesting and confirming new interesting biomarkers. Likewise, the deregulated genes might be evaluated as immunohistochemical markers, thus potentially allowing the development of immunohistochemical panels for mesothelioma.

However, the strength of this study consisted in the use of a group of genes rather than single ones for the differential diagnosis of MPM. The individuation of specific gene expression patterns of MPM could overcome the diagnostic issues related to its heterogeneity.

In conclusion, we defined two classifiers from a panel of genes, whose expression profile together with the USC classification algorithm constitutes an innovative diagnostic instrument which could be applied in the clinical routine of MPM. Obviously, further retrospective and prospective validation on a larger series of samples is needed, paying particular attention to selection of samples, since the older is the sample the lower is the RNA quality. Furthermore, the effectiveness of this diagnostic tool should be evaluated also on cytological specimens from pleural effusions, where the differential diagnosis of MPM may be difficult or even impossible [10].

In spite of the fact that this is a preliminary study, this research could allow a better pathological discrimination of epithelioid MPM and MH.

## MATERIALS AND METHODS

### Samples

This work was conducted retrospectively and it conforms to the principles of the Helsinki Declaration of 1975. In the first part of this study 36 patients with epithelioid MPM (26 males, 10 females, age ranged from 43 to 85 years, average age of 67,05 years;) and 17 with MH (13 males, 4 females, age ranged from 18 to 85 years, average age of 48,5 years) were included, in the second part 14 pleural mesothelial samples, comprising 9 MPM (6 males, 3 females, age ranged from 41 to 80 years, average age of 63,8 years) and 5 MH (3 males, 2 females, age ranged from 27 to 79 years, average age of 42,5 years) were analysed in a blind way. Informed consents were obtained from patients.

All the MPM enrolled patients consecutively underwent pleurectomy/decortication (P/D) at the Unit of Thoracic Surgery of the University of Pisa, from 2012 to 2015. Concerning the other patients, MH was an incidental finding associated with pleural inflammatory effusions and bullous emphysemas.

**Figure 4:**
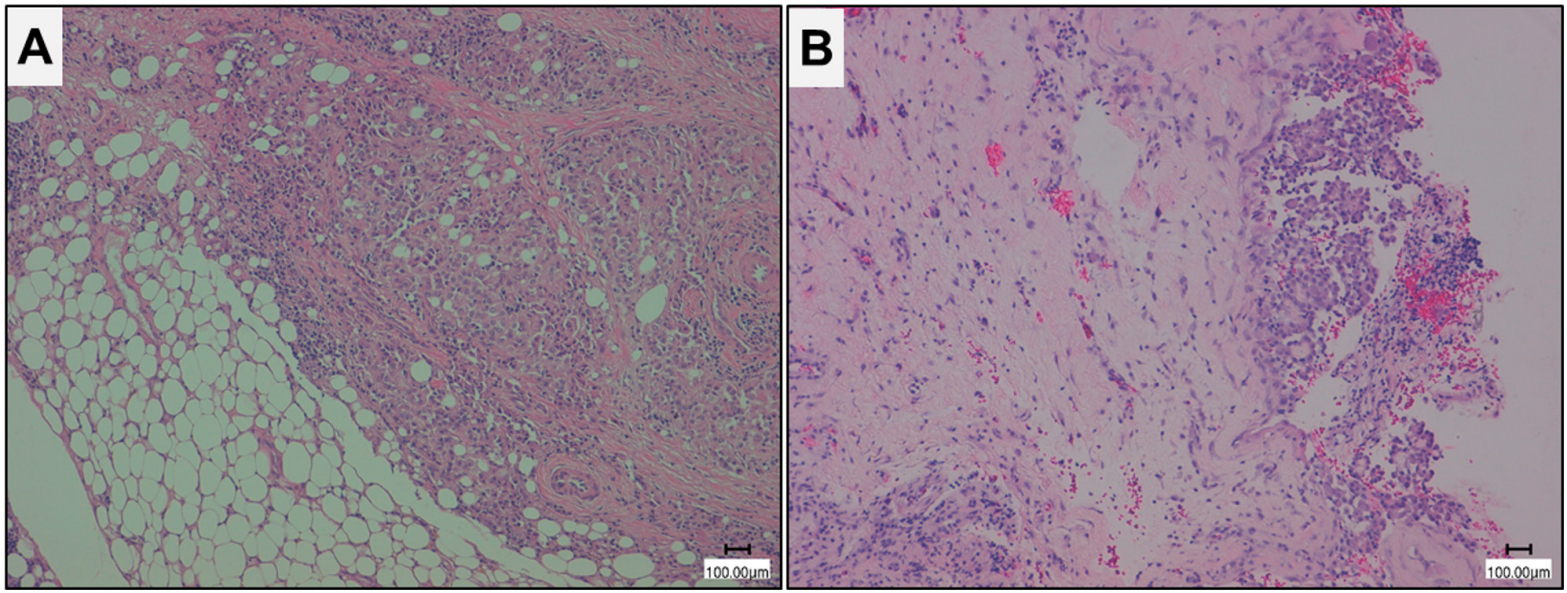
Histological imagines of representative epithelioid malignant pleural mesothelioma (MPM) and mesothelial hyperplasia (MH) **A.** epithelioid MPM; **B.** MH (Hematoxilin eosin stain, magnification 10X, scale bar 100 μm).

All tissues were formalin-fixed and paraffin-embedded and hematoxylin and eosin stained sections were prepared for microscopic examination (Leica DMD108, Leica Microsystems, Wetzlar, Germany) (Figure 4). The diagnoses of MPM and MH were independently reviewed by two pathologists (G. Alì and G. Fontanini) according to the WHO 2015 histologic and immunohistochemical criteria [7, 11].

Moreover, the most representative paraffin blocks for each sample were selected for gene expression analysis, and only the samples with sufficient tumour material (>60% tumour cells) and minimal contamination by benign cells (< 20%) were included in this study. Clinical information, including patient gender and age, was collected for each patient.

In addition, a pool of MH samples and a pool of MPM samples were analysed in each experiment as technical controls.

### RNA purification

For each sample four FFPE tissue sections, with a thickness of 5 μm, underwent standard deparaffinization and enrichment by manual microdissection. Total RNA was isolated using Qiagen RNeasy FFPE kit (Qiagen, Hilden, Germany) according to manufacturer's instructions.

The concentration of total RNA was assessed using a Xpose spectrophotometer (Trinean, Gentbrugge, Belgium). The RNA resulted adequate for gene expression analysis whenever its concentration was ≥ 30 ng/μL and its quality was acceptable if the ratio between the value of absorbance (A) at 260 nm and the one at 280 nm was ≥1.9, and the ratio between the value of absorbance (A) at 260 nm and the one at 230 nm was ≥ 2.

### nanoString ncounter analysis

The nCounter custom codeset consisted of 123 reporter and capture probe pairs directed against 117 target genes and 6 housekeeping genes for reference (Table 1). Moreover, the codeset included probes for 6 spike-in positive controls (POSi) (in vitro transcribed RNA molecules, pre-mixed with the reporter codeset during manufacturing) and for 8 spike-in negative controls (NEGi), which were not included in the reaction mix.

The nCounter custom codeset was synthesized by nanoString Technologies (nanoString Technologies, Seattle, Washington).

The RNA was hybridized using 150 ng of total RNA in addition to the capture and reporter probes in each reaction. Hybridization was performed for 18 hours at 65°C in a SensoQuest thermal cycler (SensoQuest, Gottingen, Germany). The clean-up of samples and counts of digital reports were performed as described by the manufacturers, respectively on the prep station and on the digital counter nanoString systems (nanoString Technologies, Seattle, Washington).

### nanoString data normalization

Initially, the background noise was estimated for each sample on the basis of the 8 negative control probes, and it was subtracted from the investigated gene counts in order to determine true counts.

Then, raw nanoString counts of each gene were subjected to a technical and a biological normalization, using the nSolver Software version 2.5 (nanoString Technologies, Seattle, Washington). The technical normalization allows a good control of the variability unrelated to samples, it was performed using the 6 POSi. For each sample a positive control scaling factor was calculated. If the calculated positive control scaling factor was outside a range of 0.3-3, it indicated technical problems, implicating the exclusion of the sample from further analysis. The biological normalization, on the other hand, corrects for differences in RNA input among the assays, allowing the adjustment of gene counts on the basis of reference genes. For each sample a biological normalization factor was determined and whenever it was outside the range of 0.1-10.0, the sample was excluded from the analysis. All the normalization steps were performed according to the manufacturers’ instructions (nanoString Technologies, Seattle, Washington).

### Statistical analysis

Gene expression data from the first series of samples (25 MPM and 15 MH) were subjected to a 2-way unsupervised HCA, applied independently to the samples and to the genes, using the nSolver Software version 2.5 (nanoString Technologies, Seattle, Washington). The clustering analysis was based on the Pearson correlation coefficient.

The differential gene expression between benign and malignant conditions was determined by applying the non-parametric Mann–Whitney U-test with a linearity correction, and a Spearman's correlation test was executed between age and gene expression levels, using the STATISTICA software version 10 (Stat Soft Inc, Tulsa, Oklahoma).

In order to predict the diagnostic categories of samples from their gene expression profiles we used the Uncorrelated Shrunken Centroid (USC) algorithm [38, 39]. The USC uses a shrunken centroid algorithm, based on the nearest centroid approach. This algorithm is then improved by the analysis of the interdependence of genes and by the removal of the highly correlated ones. A gene is considered to be predictive of a class if at least one of its class centroids significantly differs from the overall centroid by more than one standard deviation, and samples are assigned to a class considering the nearest average centroid pattern. The USC has two analytical phases: a training phase, using samples for which the diagnostic classes are known, and a test phase, using samples for which the classes are unknown, which have to be classified by the algorithm on the basis of gene expression levels. Our training set consisted of a series of known samples (25 MPM and 15 MH) which were perfectly representative of their own categories, and as test set we used all the 14 pleural mesothelial lesions, analysed in a blind way.
